# Potential Causes of Titanium Particle and Ion Release in Implant Dentistry: A Systematic Review

**DOI:** 10.3390/ijms19113585

**Published:** 2018-11-13

**Authors:** Rafael Delgado-Ruiz, Georgios Romanos

**Affiliations:** 1 Department of Prosthodontics and Digital Technology, School of Dental Medicine, Stony Brook University, New York, NY 11794, USA; 2 Department of Periodontics, School of Dental Medicine, Stony Brook University, New York, NY 11794, USA; georgios.romanos@stonybrookmedicine.edu; 3 Department of Oral Surgery and Implant Dentistry, Dental School, Johann Wolfgang Goethe University, 60323 Frankfurt, Germany

**Keywords:** dental implants, titanium particles, wear, corrosion

## Abstract

Implant surface characteristics, as well as physical and mechanical properties, are responsible for the positive interaction between the dental implant, the bone and the surrounding soft tissues. Unfortunately, the dental implant surface does not remain unaltered and changes over time during the life of the implant. If changes occur at the implant surface, mucositis and peri-implantitis processes could be initiated; implant osseointegration might be disrupted and bone resorption phenomena (osteolysis) may lead to implant loss. This systematic review compiled the information related to the potential sources of titanium particle and ions in implant dentistry. Research questions were structured in the Population, Intervention, Comparison, Outcome (PICO) framework. PICO questionnaires were developed and an exhaustive search was performed for all the relevant studies published between 1980 and 2018 involving titanium particles and ions related to implant dentistry procedures. Preferred Reporting Items for Systematic Reviews and Meta-Analyses (PRISMA) guidelines were followed for the selection and inclusion of the manuscripts in this review. Titanium particle and ions are released during the implant bed preparation, during the implant insertion and during the implant decontamination. In addition, the implant surfaces and restorations are exposed to the saliva, bacteria and chemicals that can potentially dissolve the titanium oxide layer and, therefore, corrosion cycles can be initiated. Mechanical factors, the micro-gap and fluorides can also influence the proportion of metal particles and ions released from implants and restorations.

## 1. Introduction

Titanium implants have been used for dental, orthopedic and other medical applications since the early 1980s [1,2]. Implant-related factors, such as the surface characteristics, material composition and chemistry, are responsible for the osseointegration. Specifically, the presence of a titanium oxide layer on the implant surface is considered crucial for the maintenance of the osseointegration and the prevention of the corrosion of the titanium surface [3]. The dental implant surface does not remain unaltered and, if changes occur at the implant surface, mucositis and peri-implantitis processes could be initiated; osseointegration might be disrupted, and bone resorption phenomena (osteolysis) may lead to implant loss [1,2,3,4]. Particularly, at the moment of implant insertion, the implant surface can incur changes in its chemical and topographic structures, which is sometimes irreversible [1] and also titanium particles of different sizes and characteristics can be released from the dental implant surface [5,6,7,8,9].

Subsequently, if the implant surface is altered, degraded or dissolved by the effects of acidic substances or an acidic environment, the titanium oxide layer can be lost, and corrosion phenomena can begin [10,11,12,13,14]. Furthermore, during functional loading, the combination of mechanical and chemical corrosion (fretting) between the implant surface and the adjacent bone can facilitate the release of more metal particles and ions and, if friction occurs between the internal implant walls and the prosthetic abutment, additional particles and ions can be released into the surrounding tissues [2,3,4,15]. Lastly, cleaning and disinfection of the implant surface with mechanical, physical or chemical methods have been shown to induce changes of different magnitudes at the implant and abutment, with subsequent titanium oxide layer loss, titanium particle or ion loss and corrosion initiation [16,17,18,19,20].

However, the sources of titanium particle or ions in implant dentistry procedures have not been studied in depth and methods for its reduction are currently unknown. Therefore, all the information related to the potential sources of titanium ions and particles and suggestions of methods for their control in implant dentistry is fundamental for the long-term survival of dental implants to improve clinical practice.

The purpose of this systematic review is to compile the information related to the potential sources of titanium particles and ions in dental implantology. Taken together, the findings of this review, including the percentages of particles, their characteristics, and detection methods, suggest ways for reducing titanium particle and ion release in implant dentistry.

## 2. Results

**Figure 1:**
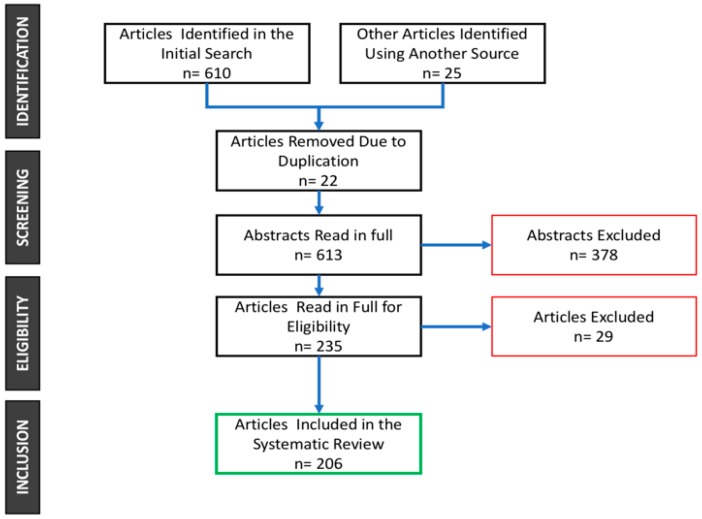
Preferred Reporting Items for Systematic Reviews (PRISMA) flow diagram of the screening and selection process.

The initial search returned 635 articles. A total of 22 articles were removed because they were duplicates; the remaining 613 articles abstracts were read in full, and 378 articles were excluded because they did not fulfil the inclusion criteria. The full texts of the remaining 235 articles were read in full for eligibility, and 29 articles were removed because they presented reviews or expert opinions or were duplicated. Finally, 206 articles were included in this systematic review (Figure 1).

**Figure 2:**
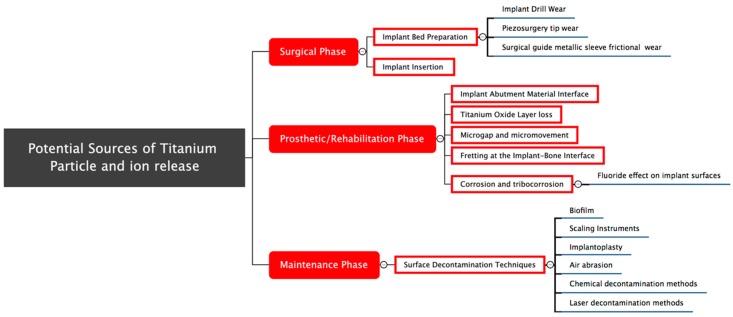
This systematic review found that titanium particles and ions can be released during the surgical, prosthetic and maintenance phases due to different causes during the life span of a dental implant. The rectangles filled in red are the three phases in implant dentistry procedures in which titanium particles and ions can be released. The rectangles with the red frame are procedures within the previous phases which resulted in titanium particles and ions release. The underlined sentences are the detailed sources or initiators of titanium particles and ions release.

The findings were grouped as follows: sources of titanium release during the surgical phase, during the prosthetic phase and during maintenance (Figure 2).

### 2.1. Potential Causes of Titanium Particle Release during the Surgical Phase

#### 2.1.1. Implant Bed Preparation

Bone-cutting instruments, when subjected to frictional forces, can suffer variable levels of metal attrition, wear and corrosion. Traces of different metallic elements have been observed after osteotomy in regional ganglions, kidneys and lungs [21,22]. In addition, it has been demonstrated that the irrigation liquid collected from the implant bed preparation contains metallic debris and ions. Rashad et al. [23] performed implant osteotomies with rotatory instruments and piezosurgery devices to detect drill deposits in bone or in the recovered irrigation liquid. After implant bed preparation, bone samples were examined by (Scanning Electron Microscope) SEM/energy dispersion X-ray spectroscopy (EDX), the irrigation liquid was collected and filtered with polycarbonate membranes with a specific pore size. The membranes were further analysed by SEM/EDX for the detection of metal content. The results showed different residual metals from the drills in the irrigation liquid (Ag, Si, Fe, Ti, V, Cu, Mn, Zr, Cr, Bi, Mg) for both osteotomy methods. The authors recommended the use of copious irrigation to reduce the metallic particle content in the implant bed area [23].

Implant drill bits and piezosurgery tips suffer characteristic patterns of wear after osteotomy procedures. Drills showed abrasive wear, plastic deformation, blunting, coating damage and material loss, mainly at the tip and cutting edges of the drill flank [24]. Meanwhile, piezosurgery showed abrasive wear of the tip and flanks and plastic deformation of the cutting point. The characteristics of the wear suffered by osteotomy instruments have been related to the drill material, drill and drill mechanical properties [24].

Carvalho et al. [25,26] demonstrated that substance loss, steel melting and condensation of particles detached from the active point increased proportionally to the number of uses; indeed, drills used 10 times showed 17.86% deformation and, drills used 50 times showed 33.97% deformation. These findings were also confirmed in drills used 50 or more times, on which increased areas of metal subtraction, the addition of loosened metal, and metal surface abrasion at the lateral surface of the cutting tip and drill edges were detected [27,28,29].

Another factor that seems to increase the wear of implant drills is the use of guided surgery techniques. Bone heating, drill deformation and roughness were evaluated after osteotomy with guided surgery and conventional osteotomy; greater drill deformation and wear were recorded after the 40th osteotomy in the guided surgery group produced by the drill friction against the metal sleeve and the bone [30].

Sterilization and irrigation also increase the release of metal particles and ions as well as drill wear. Allsobrook et al. [31] evaluated Straumann, Nobel Biocare and Neoss drills. The authors performed 50 osteotomies per drill. After 20 osteotomies, the drills suffered 30 µm to 100 µm of wear at the tip and edges and loss of the surface coating. Furthermore, analysis of corrosion after sterilization processes and irrigation with saline showed increased pitting corrosion of the drills after 20 cycles of sterilization [24,31,32]. Other authors also found that the autoclave sterilization of implant drills increased the surface corrosion of the drills and therefore the ion and particle release [25,33,34]. Similar results were obtained when implant drills with three different characteristics (smooth stainless steel drills, coated drills and smooth zirconia drills) were compared. After repeated drilling and sterilization, it was found that drills in all groups suffered wear, deformation, coating delamination and surface roughness changes [35].

The material of the drill seems to influence the amount of particle loosening of the drill surface. Zirconia drills showed less qualitative substrate loss than titanium drills when drilling 20 implant beds at a standard drilling speed of 800 rpm [36].

##### Remarks

**Figure 3:**
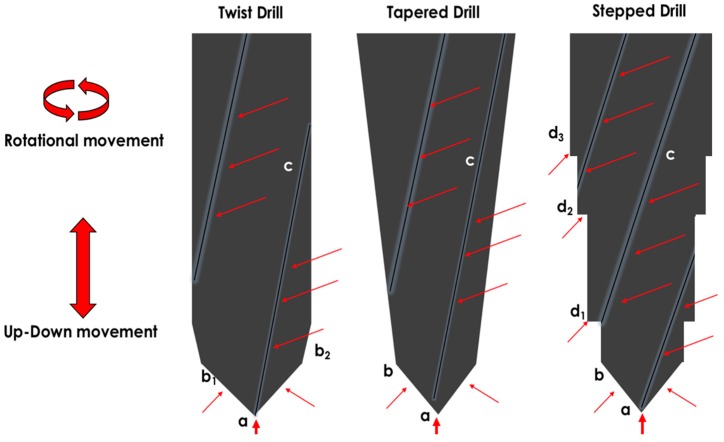
Areas of wear of implant drills. Different drill designs present wear and particle loosening at different levels under the effects of axial and rotational forces. Twist drills suffer deformation, blunting and delamination of the drill tip (a), tip angles (b1 and b2), and cutting blades (c). Tapered drills suffer deformation, blunting and delamination of the drill tip (a), tip angle (b), and cutting blades (c). Stepped drills suffer deformation, blunting and delamination of the drill tip (a), tip angles (b), cutting blades (c) and step angles (d1, d2, d3). Thin red arrows are showing the blade areas suffering wear as well as the tip angles. Thick arrows are showing the tip of the drills suffering wear and deformation.

**Figure 4:**
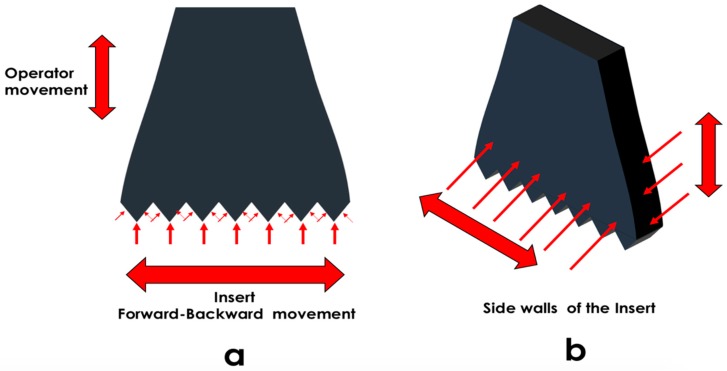
Areas of wear of piezosurgery inserts. The piezosurgery insert directions of movement: a vertical movement induced by the operator, and a minimal vertical displacement during the ultrasonic movement in conjunction with a horizontal component produced by oscillation of the tip. (**a**) The piezosurgery insert oscillates in a forward-backward movement. The insert tip and the sides of the tip suffer deformation, wear and particle detachment. (**b**) Additionally, the sidewalls and borders of the insert suffer deformation, wear and particle detachment. Short and long thing arrows are showing areas of angle and lateral wear and deformation. Thick arrows are showing the vertex of the tips suffering wear and deformation.

**Figure 5:**
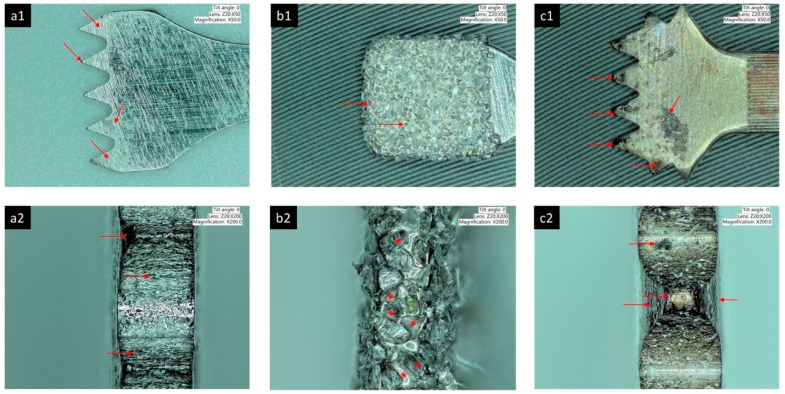
Piezosurgery tips used 1 time. The tips are made from different materials. (**a1**,**a2**) Stainless steel insert showing lateral wear of the active tip, abrasion and particle loosening at the flanks, deformation of the tip edges and material delamination. (**b1**,**b2**). Diamond-coated insert showing wear at the sides and empty spaces representing particle loosening. (**c1**,**c2**) Gold nitride-coated insert with several areas at the flanks, tips and sides of the tips showing excessive wear, particle delamination and deformation. The red arrows are showing the areas of wear, delamination and deformation suffered by piezosurgery inserts. The red asterisk is showing particle detachment from diamond coated piezosurgery inserts.

During osteotomy procedures, surgical drills, implant drills and piezosurgery tips suffer deformation, surface wear, microfractures, delamination, and metal particle release (Figure 3, Figure 4 and Figure 5). Additionally, sterilization of the cutting tools can initiate corrosion and can increase the particle and ion release. Sufficient irrigation, adequate suction of metallic debris, the use of single-use drills, adequate control of the number of sterilization procedures, inspection of cutting tool integrity, timely replacement of worn drills and the use of harder and more resistant cutting tools might reduce the deposition of metallic particles and ions released during implant bed preparation.

#### 2.1.2. Implant Insertion

When the dental implant is inserted, frictional moments occur at the bone-implant interface and the initial mechanical interlocking results from interactions between the bone and the implant threads and walls [37]. As a consequence of implant insertion, microfractures and compression occur at the bone side and the implant surface is simultaneously subjected to a combination of torsional and frictional forces, which may alter the original implant surface. Wawrzinek et al. [38] described that shear forces originating from the friction of implants inserted against the surrounding bone tissue can produce a shifting of stresses at different locations along the implant surface related to the heterogeneity of the bone tissue (cortical or cancellous) and the implant geometry. Therefore, localized areas of stress concentration can appear and titanium particles can be released into the bone tissue [37]. A harder implant surface should be able to retain its characteristics more than a softer surface and should demonstrate increased abrasion resistance [39], however, the abrasive wear phenomena during implant insertion are influenced by additional factors that cannot be controlled simultaneously; these factors include the properties of the specimens, interactions with the environment and the experimental conditions [40,41,42].

Schliephake et al. [43] showed that the placement of self-tapping titanium implants abraded particles from the implant surface, as evidenced by deposits of these particles at the implant-bone interface. Seki et al. [44] and Kim et al. [45] revealed titanium particles in the tissue interposed between the bone and the titanium plate used for oral and maxillofacial fracture fixation. The authors speculated that the particles originated from two causes: first, the physical-mechanical removal of the oxidation layer during insertion; and second, the dissolution of the subjacent titanium into the surrounding tissues (corrosion), resulting in detection of the titanium particles and ions [44,45].

Different observations also showed that particles abraded from the implant surface ranged between 1.8–3.2 µm in diameter and were located at the bone surface and up to 100 µm inside the surrounding bone [5]. Other authors found titanium particles ranging widely in size from 20 nm to 20 µm at the implantation site concentrated at the cortical layer. Apparently, the process of implant insertion alone can release up to 0.5 mg of metallic debris at the implant-bone interface. Previous studies in orthopaedics have shown that aseptic osteolysis can be induced by 0.220 mg to 3.0 mg of titanium particles in the implant area as well as inside the medullary spaces [6]. In the case of titanium plasma-sprayed (TPS) implants, the particle dimensions decreased with increasing distance from the implant surface, probably due to gradual and passive dissolution, fretting and wear [7,8,9].

Martini et al. [46] found that titanium particles released from TPS implants can be present within 200–250 µm of the implant surface and some debris could be observed at 500 µm. It was speculated that detachment of titanium particles from the TPS implants resulted from the implant surface morphology, frictional forces during implant insertion and frictional forces between the titanium (Ti) coating and the pre-existing bone. These released metallic particles impeded bone formation and created gaps of 180–260 µm between the pre-existing bone and implant surface compared to non-TPS surfaces, which showed bone matrix on the implant surface after 14 days [47,48,49].

Wennerberg et al. [50] performed in vitro and in vivo studies to investigate the titanium concentration around titanium implants with different surface roughness inserted in rabbit tibias. They found that moderately rough surfaces (S*a* 2.21 µm) presented more titanium release up to 400 µm from the implant surface than smooth surfaces (S*a* < 1.43 µm). The authors recommended further chemical analysis of the implant surfaces to evaluate potential chemical changes at the implant surfaces and their biological consequences [51].

Mints et al. [52] compared acid-etched, anodized and machined implant surfaces after their insertion into bone blocks. They found that all the implants suffered surface damage, material removal from the implant surface, oxide layer breakdown, metal debris transportation (due to the insertion and friction) from the apical and middle third to the coronal area and cracks at the implant surface as result of the insertion process. The titanium debris ranged from nano- to microparticles; the nanoparticles were located in the coronal area and the microparticles were located in the mid and apical regions [52].

It seems that although almost all of an implant surface is exposed to wear and particle detachment during insertion, the tip of the threads and the lower flank of the threads are more exposed, while the apex microstructure is least exposed in conventional implant bed preparation [53]. It was also concluded that surfaces with subtractive modifications appeared to suffer less wear and particle loosening than surfaces with additive modifications and that re-establishment of the damaged TiO_2_ layer was superior for surfaces with subtractive modifications [54].

Particles and ions released during titanium implant insertion can also be retained at the soft tissue level. Concentrations of specific metallic elements retained in the gingival cuff, surrounding the implant neck were detected in an analysis of histological sections processed with the laser ablation detection technique. The elemental mapping showed titanium from 0.4 mm up to 4 mm from the implant, with additional contents of aluminium and vanadium [55].

One in vitro study did not find metal particles released after the insertion and removal of sandblasted and acid-etched implants. The study was performed in polyurethane blocks with different densities and the methods of analysis were SEM observation of the implant surface and X-ray diffraction analysis. Albeit authors observed surface deformation, changes and wear, no traces of metals were observed in the samples [56]. Titanium particles and ions are not always detected; apparently, the irrigation of the surgical site could dilute and remove metallic particles and ions, thus reducing the overall metal content. However, the presence of measurable metals and ions in intraoperative fluid samples indicates that metal particles and ions are certainly released at the time of implantation [57].

The Food and Drug Administration (FDA), explained that variability in metals detection is possible given test interpretation differences, accuracy and precision of the detection method, variability in the test specimen, contamination by metal ions, variance between laboratories, interfering substances and lack of proficiency testing [58,59]. These factors should be considered in future comparisons of experiments.

##### Remarks

Implant insertion produces changes at the implant surface, as one or more of the following factors were observed: wear, deformation, particle delamination, scratches and cracks at the lower flank of the threads, thread tips and implant apex in different proportions. Released metallic particles with variable sizes and metallic ions can be located adjacent to the implant surface or can be displaced to other distant locations (Table 1). Factors, such as higher bone density, additive implant surfaces and lack of irrigation might lead to the detection of higher percentages of metallic particles and ions, while lower bone density, subtractive implant surfaces, and abundant irrigation might reduce the number of detectable metallic particles and ions produced during implant insertion. It is recommended to standardize the methods of metals particle and ion detection, as suggested by the FDA [59].

### 2.2. Potential Causes of Titanium Particle and Ion Release in the Prosthetic Phase

The implant-abutment interface involves the interaction of the internal walls of the implant connection and the surface of the abutment connection. The material properties, the magnitude, direction and duration of the forces, the composition and saliva pH and the microflora can all influence the amount of titanium particles and ions released from the implant-abutment interface into the surrounding environment [61,62,63,64,65,66,67,68,69,70].

#### 2.2.1. Implant-Abutment Material Interface

Titanium and zirconia abutments induce different levels of wear to the implant connection. Klotz et al. [61] applied cyclic load of up to 1,000,000 cycles with forces from 20 N to 200 N to titanium and zirconia abutments connected to titanium implants. They found that implants with zirconia abutments suffered greater wear and more titanium particle release than implants with titanium abutments. The authors explained that these differences were produced because the hardness of the zirconia is approximately 10 times higher than the hardness of grade 4, commercially pure titanium (1600–2000 Vickers hardness (HV) for zirconia vs. 258 HV for titanium) [61,66,67].

The amount of wear at the implant-abutment connection was quantified by Stimmelmayr et al. [62], who demonstrated significantly more wear at the shoulder of implants connected to zirconia abutments (10.2 ± 1.5 µm) than that of implants connected to titanium abutments (0.7 ± 0.3 µm) [62]. When materials with different mechanical properties interact, more wear and deformation are expected in the weakest material. Indeed, the zirconia flexural strength is greater than 1000 MPa, and its elastic modulus is greater than 200 GPa, making it a more rigid material than titanium [63,64].

In similar studies comparing the zirconia abutment-titanium implant interface with the titanium-titanium interface, small regions of scratching and crushing after dynamic loading were observed in the titanium-titanium interface. In contrast, the zirconia-titanium implant interface was dramatically affected after dynamic loading. The interface between materials with very different Young’s moduli could suffer wear, micro separations, and consequently, mechanical failure of the connection [65].

Furthermore, interactions between pure titanium and titanium alloys with greater hardness can result in more deformation and wear at the implant connection. For instance, an abutment made in titanium alloy (Ti6Al4V) is characterized by a mean hardness value of approximately 350–370 HV, while an implant synthesized from commercially pure titanium has a hardness of approximately 200–280 HV. Consequently, plastic deformation and wear often occur within implant connection surfaces, resulting in loosening of the joint and a reduction in its mechanical integrity [68,69,70].

##### Remarks

Implant-abutment connection wear is influenced by the characteristics of the coupled materials. The combination of an implant with an abutment (harder or softer) will result in wear of the weakest involved material and potentially in metallic particle release. The released particles can remain inside the connection area (thus increasing the frictional wear) or can be displaced to adjacent tissues, potentially favoring a foreign body reaction.

It is recommended to utilize materials with similar hardness and mechanical properties to reduce the wear of the abutment and the inner walls of the implant at the implant-abutment connection.

#### 2.2.2. Microgap and Micromovement

The mismatch between implant and abutment components (micro-gap) can be increased by micromotion phenomena under functional loading, which could result in increased friction, microleakage, material wear, titanium particle release and screw loosening [68,71,72,73,74,75,76,77,78,79,80]. Braian et al. [71] evaluated the horizontal micro-gap between abutments and implants with external and internal hexagonal connections. They found the smallest micro-gap for prefabricated gold abutments (less than 50 μm) placed on implants with external hexagon compared to prefabricated plastic cylinders (less than 130 μm) placed on implants with internal hexagonal connections.

Meanwhile, Morse taper connections with no or minimal micro-gap and titanium abutments instead of zirconium abutments can reduce the wear and micromovement at the implant-abutment interface [72,73,74]. Conical connections also showed micro-gaps. Fatigue changes of conical connections were evaluated after functional loading by SEM and EDX. The results showed that the micro-gap between dental implant-abutment assemblies with conical connections exists prior to cyclic loading, that titanium and metallic particles (ranging from 2 µm to 80 µm in diameter) were released within the interface and outside the interface and that the effects of the wear and the micro-gap increased with the number of cycles [75].

The physicochemical and microscopic characteristics of different implant-abutment configurations were evaluated, and without exception, defects from 0.5 to 5.6 μm were present in all the samples. Therefore, the original micro-gap between the parts can lead to micromotion and wear, the release of more particles, the penetration of oral fluids and bacteria into the connection, the initiation of corrosion, as well as potential late failures [68,76,77,78,79,80].

##### Remarks

The micro-gap potentially exists in all implant-abutment connections, given unavoidable discrepancies in the fabrication process. Its dimensions are variable and it has been demonstrated that smaller micro-gaps are present in Morse and conical connections and that larger micro-gaps exist in external connections than internal connections. Larger micro-gaps result in increased micromovements, which under functional loading can increase the friction between the parts, the wear and the particle release. In addition, under functional loading, saliva and bacteria will flow, producing an additional effect, along with the displacement of titanium particles to peri-implant tissues and peri-implant bacteria to the gap of the implant-abutment connection. The presence of saliva, bacteria and their sub-products and the wear and the corrosion initiation of the metallic parts inside the implant-abutment connection might induce mechanical (screw loosening, corrosion, fatigue, fracture) and biological (mucositis, peri-implantitis) implant failures.

Therefore, it is recommended to use internal connections and Morse or conical connections that possess smaller micro-gaps. Additionally, implant companies should provide information about the micro-gap that exists in their implant-abutment connections to clinicians.

#### 2.2.3. Titanium Oxide Layer Loss

When the titanium implant surface is exposed to air, a stable titanium oxide film is spontaneously formed at the implant surface. This thin layer (1.5–10 nm thickness) is formed due to the high affinity of Ti for oxygen [81,82]. The oxide layer protects the bulk material from reactive species and consists of TiO_2_ coexisting with other titanium oxides, such as TiO and Ti_2_O_3_ [83,84]. The resistance to corrosion of titanium implants originates in this titanium oxide layer [85,86,87].

Once the oxide layer is formed (passivation), it can be altered and damaged by various environmental and functional factors and in the event of damage, the oxide layer can spontaneously reform under normal physiological conditions (re-passivation) [86]. Examples of environmental and functional factors that can alter the oxide layer are abnormal cyclic loads (overloading or continued loading), micromotion of the implant, micromovement of the implant-abutment interface, acidic environments and the combined effects of these factors [88]. Continued attack of the implant surface by these factors can result in permanent breakdown of the oxide film, leaving exposed the bulk metal to electrolytes. Varying pH conditions can turn the implant environment into a more acidic environment and active dissolution of metallic ions can occur (corrosion) [89,90,91].

Regarding the effects of an acidic environment on the integrity of the titanium oxide layer and bulk titanium, metabolites, such as lactic acid can induce the depletion of oxygen sources required for re-passivation, thus hindering the capability of the titanium surface to reform the oxide layer [11,89,91,92,93,94,95,96,97]. Therefore, metallic Ti ions are released into the surrounding tissues. Simultaneously, metallic debris is also loosened from the weakened implant surfaces exacerbating inflammatory conditions and facilitating further surface corrosion [11,89,91,92,93,94,95,96,97].

Saliva also plays a role in the corrosion of dental implants [98]. The dental implant interface is exposed continuously to saliva via the gingival sulcus. Saliva can act as a weak electrolyte, and the oral cavity can simulate an electrochemical cell facilitating dissolution of the oxide layer. Further electrochemical corrosion of titanium and its alloys may lead to crevice corrosion and ultimately the release of corrosion products [90,99,100].

##### Remarks

The titanium oxide layer appears on the implant surface as soon as the implant comes into contact with air, and although the titanium oxide layer has the capability to regrow (re-passivation), the action of continued wear, exposure to chemicals, bacteria and their sub-products, and the presence of an acidic environment can deteriorate and degrade the titanium oxide layer. To preserve the oxide layer integrity, the use of non-aggressive chemicals to disinfect the titanium surface and reduce the bacterial content and environmental acidity is recommended.

#### 2.2.4. Corrosion

Degradation of the implant surface in the human body can be induced by two main events, i.e., wear (a mechanical degradation producing particles) and corrosion (a chemical degradation that mainly produces soluble metal ions) [101]. The term “corrosion” is generally used for metals and consists of material degradation induced by actions of the environment [102,103,104].

Over time and by the effects of implant function, the implant surface can experience corrosion and can release ions and particulate debris [4,105]. The metallic debris released from titanium implants can be present in various forms, including nanometric and micrometric particles, colloidal and ionic forms (bonded to proteins) [106,107], organic forms (iron-storage complexes), inorganic metal oxides and salts [106].

In the oral cavity, fluctuations in temperature, pH, oxygen, bacteria and food decomposition are attacking continuously the implant surface [91]. The TiO_2_ layer is disrupted, and ions and debris generated by the physicochemical degradation of the surface are removed [108]. Through the abrasion produced by food, liquids and toothbrushes, the cycle continues [109].

Factors that can alter the corrosion resistance of the titanium surface are inflammation of the surrounding tissues (which can produce local acidification) and the acidic environment created by lactic acid released by bacteria [94,110]. The lipopolysaccharide (LPS) of gram-negative bacteria can increase the inflammation of peri-implant tissues by its marked effects on macrophages, lymphocytes, fibroblasts, and osteoblasts [111,112]. LPS chains attack the oxide film of the titanium surface (by adsorption phenomena), inducing voids in the oxide film. The Ti surface is exposed and ion exchange between the exposed surface (metal ions, M^+^) and the saliva (electrons, e^−^) initiates the corrosion process [113].

Furthermore, the interactions between the current flows of the dental implant and the prosthetic superstructures (produced by the differences in the electric potential of the materials) can create crevice, pitting and galvanic corrosion and the subsequent dissolution of the pure metal and alloy components [100]. Under mechanical loading, the corrosion resistance of the metal alloy decreases in different proportions, with cast and machined titanium having the most passive current density at a given potential and chromium-nickel alloys having the most active critical current density values. High-gold-content alloys have excellent corrosion resistance, and palladium alloys have a low critical current density due to the presence of gallium [114].

Chromium-cobalt framework and implant interactions were analysed in vitro, and the results showed that both the implants and the frameworks suffered active degradation processes, ions of all the materials were released and leakage of cobalt ions was greater than the leakage of titanium and chromium ions. In addition, the surfaces of the implants and frameworks became rougher after exposure to saliva [115]. These differences in percentages could be explained by the nature of metallic elements (crystalline structure, surface energy, solubility and exposed area) [116,117] and the quantity and duration of exposure [118]. Ion leakage will occur for all solid surfaces in contact with liquids until the solubility constant is reached [93].

Corrosion could occur also at the intraosseous portion of the implant surrounded by bone. This occurs by a phenomenon in which two surfaces (the implant surface and bone) under mechanical loading can have an oscillatory relative motion of a small, amplitude (fretting) [93], in which chemical reactions are prevalent. This type of corrosion is characterized by particle removal, oxide formation and increased abrasion, which increase the wear of the implant surface [119].

#### 2.2.5. Tribocorrosion

This mechanism involves a combination of tribological (wear and fretting) and corrosive (chemical or electrochemical) events and is influenced by variations in mechanical contact conditions (loading and relative velocity) and in the nature of the environment (pH, humidity and biochemistry) [120,121,122].

Revathi et al. [120] described the sequence of tribocorrosion as follows: a given load is applied between two surfaces; in the presence of lubricant particles, the load will allow a sliding movement that will produce oxide layer fractures, microcracks, diffusion and re-passivation, wear debris release and finally material dissolution through five types of corrosion, i.e., microbial, galvanic, uniform, crevice and fretting corrosion [121].

Specifically, for titanium implants and the salivary pH, the titanium showed inferior performance in tribocorrosion at pH 6.0, which manifested as greater weight loss and increased cracking [113]. The normal pH of saliva ranges between 6.3 and 7.0 [11,123], various conditions can lower the pH of saliva to below 6.0 (infection, food, oral hygiene products, age, periodontitis, smoking, systemic disease and salivary gland radiation) [123], favoring the corrosion process [124,125,126]; under these simultaneous actions, the total material loss may be significantly greater than that under mechanical wear or corrosion individually [127].

#### 2.2.6. Fluoride and Titanium Corrosion

Fluoride is one of the main methods for dental caries prevention and is present in many toothpastes and gels. Its percentages range from 0.1 to 2.0 wt%. At these concentrations, fluoride can reduce the corrosion resistance of metallic implants [128]. Apparently, the presence of fluoride ions in the electrolytic environment of the oral cavity attacks the titanium oxide layer and facilitates its dissolution [129].

The interaction between fluoride and titanium surface was described by Kaneko et al. [130]; the oxide film reacts in the presence of fluoride solutions by forming complexes of molecules on the implant surface (titanium fluoride, titanium oxide fluoride and sodium titanium fluoride). These soluble molecules replace the titanium oxide film, allowing corrosion initiation [130]. Once the titanium oxide film is lost, the compound films formed on the surface will undergo rapid dissolution. The rate of dissolution depends on the formation of a new oxide compound at the metal oxide interface, the flow of electrons to fill the metal or oxygen vacancies in the film, the generation or consumption of vacancies at the oxide/electrolyte interface and the chemical or electrochemical dissolution process itself [10,11,12,13].

The effect of the time of immersion of titanium implants with different surface treatments (sandblasted and acid-etched, micro-sanded with calcium phosphate and acid-etching, saline solution and saliva) on the percentage of metallic particle and ion release were evaluated by Barbieri et al. [14]. Different time points were selected, and mass spectrometry was used for the detection of particles and ions in the solution. The authors found that all implant surfaces released titanium, nickel and vanadium after seven days and that these percentages increased in proportional to the elapsed time until the sixth month [14].

##### Remarks

Fluoride ions have the capability to bond to the titanium oxide layer and create compounds that dissolve easily in acidic media, thereby facilitating titanium or metal dissolution and particle and ion release. Although the behavior of fluoride in vitro may vary slightly compared to that in clinical settings (based on the buffer capability of food and saliva), the noxious effect of fluoride on titanium corrosion cannot be denied. It is recommended to utilize non-fluoride rinses or gels for daily care and use alternative (non-acidic) substances in patients with titanium dental implants.

### 2.3. Potential Causes of Titanium Particle and Ion Release during the Maintenance Phase

The maintenance phase of dental implants and restorations involves the control of biological (i.e., biofilm and plaque) and mechanical risk factors. There are no defined protocols for dental implant maintenance, cleaning or disinfection, and apparently, all existing methods have negative effects of different magnitudes on the implant surface.

#### 2.3.1. Biofilms

The extraosseous surface of dental implants (i.e., polished neck) and restorations (i.e., abutments, metal frameworks) will develop a biofilm once exposed to the oral cavity environment [131,132]. Different surface characteristics, such as chemistry, energy and topography, can influence biofilm formation [133,134].

The first step in biofilm formation is the adsorption of a layer of organic molecules (i.e., salivary proteins) to the material surface [133]. The subsequent step is the adhesion of cells and bacteria mediated by membrane binding sites, such as glucan-binding sites or specific protein-binding sites, such as those for proline-rich proteins [134]. Then, different bacterial populations attach to the pellicle, allowing accumulation of the biofilm, which depends on additional surface characteristics, such as the surface tension. These steps were demonstrated in experimental studies showing that the initial retention of microorganisms to surfaces was strongly related to the forces required for mechanical removal and to the energy of the exposed surface [135].

Within the oral cavity, there is a continuous introduction and removal of microorganisms and nutrients; for these microorganisms to survive they must be adhered to soft or hard tissues to resist, shear forces [135,136]. Bacterial adhesion to soft tissues is reduced by the turnover of the oral epithelia, however, hard, solid structures (teeth, dentures, implants) provide non-shedding surfaces, which allow the formation of thicker and more stable biofilms [137]. In general, established biofilms maintain equilibrium with the host, but uncontrolled accumulation or metabolism of bacteria on hard surfaces can cause dental caries, gingivitis, periodontitis, peri-implantitis and stomatitis [137].

Oral bacteria will adsorb, organize and group on exposed surfaces, forming plaques. These microenvironments of bacteria and their sub-products require efficient removal from the contaminated surface. The main problem associated with plaque removal from an implant surface is potential damage to the surface, which can be permanent [138,139].

Therefore, methods of implant surface detoxification and plaque and calculus removal causing little or no damage to the surface should be preferred. Current methods used for the decontamination of implant surfaces include mechanical instruments, chemical agents and lasers [140]. Different advantages, disadvantages and limitations have been correlated with these methods, thus, there is no defined gold standard for implant surface decontamination [141].

#### 2.3.2. Scaling Instruments

Non-metal instruments (plastic and Teflon tips) were found to cause minimal damage to both smooth and rough titanium surfaces. Meanwhile, hard instruments (metallic) cause major damage to smooth and rough surfaces [16,17,18,19,20,142,143]. Burs seemed to be the instruments of choice if the smoothening of a rough surface was required, but they led to increased metal particle release [16]. Non-metal instruments and rubber cups were shown to be adequate for smooth and rough implant surfaces, air abrasives should be preferred if the surface integrity must be maintained. However, these approaches involve two events that alter the original surface: first, the release of titanium particles produced by the cleaning method; and second, the deposition of instrumentation materials, and residues of the air-abrasive (cleaning powders) [17].

Instruments used for the mechanical cleaning of the implant surface, such as metal curettes and conventional sonic and ultrasonic scalers can damage the implant surface (particle release, modification of the original surface topography and chemical changes). Meanwhile, non-metal instruments and air abrasives produce less damage and fewer alterations to the surface but have been associated with incomplete plaque removal and the generation of sub-products [16]. If the surface roughness is modified, biofilm formation or cell re-attachment can also be influenced, thus altering the healing process [17].

Hallmon et al. [18] and Homiak et al. [19] also found that after multiple uses, plastic curettes changed the structure of the titanium surface. Similarly, Cross-Poline et al. [20] studied the effects of instrumentation on titanium surfaces. They found that the surfaces changed compared to the original control surfaces and observed both particle detachment from the surface and traces of instrumentation materials on the treated surface [20].

The most external areas of the implant are more exposed to damage and deformation than internal areas during mechanical instrumentation. Augthun et al. [142] found roughening of the original implant surface at the implant thread edges after the use of a steel curette for 60 s. Differences can be expected given the multiple factors that influence the extent of surface damage, i.e., the number of strokes, the pressure, the number of treatments and the cleaning instrument [143].

#### 2.3.3. Implantoplasty

This procedure aims to reduce the adherence of plaque by eliminating the contaminated titanium surface, removing inaccessible areas below the contaminated threads, and smoothing the surface topography, thus facilitating implant cleaning [144]. The most efficient drills for implantoplasty regarding the smoothness of the surface achieved were conical carbide drills (Ra < 1 µm) compared with round carbide drills (Ra > 1 µm) [18,145].

Intentionally changing the implant surface by implantoplasty with diamond burs and polishing devices was evaluated; originally, smooth titanium surfaces suffered severe damage and increased surface roughness, together with metallic traces and deposits on the titanium surfaces [146]. When performing implantoplasty, titanium or metallic particles are released, and extraordinary care must be taken to completely remove all titanium particle deposits from the surrounding tissue [147]. Carbide and diamond burs for implantoplasty were compared in vitro; the burs were used alone and in sequence. The original roughness of titanium implants with TPS and SLA surfaces were compared with that of the surfaces after implantoplasty, and both drills changed the roughness of both surfaces [148]. Implantoplasty can reduce bacterial adhesion (through reducing the surface roughness and eliminating non-cleansable areas), but some risks exist with this treatment, including high temperatures, released particles, implant surface damage, implant diameter reduction and implant fracture [149].

Implants immersed in acrylic blocks, were treated by implantoplasty with one of the following methods: diamond burs and silicone polishers; diamond burs and Arkansas stones; diamond drill short sequence; diamond drill short sequence and silicone polishers; diamond drill complete sequence; and diamond drill complete sequence and silicone polishers [150]. The authors found that all methods reduced the surface roughness, but pollution of the operative field was observed in the groups using silicone polishers. The authors concluded that the use of diamond burs and Arkansas stones resulted in a smoother surface with less debris and recommended studies investigating the bio-toxicity of the different types of debris that can be generated during implantoplasty procedures [150].

One potential drawback of implantoplasty procedures is that titanium particles removed from a contaminated implant surface are also contaminated and may not be fully removed from the surrounding environment, which could disseminate bacteria into the surrounding tissues [151].

##### Remarks

Implantoplasty is the implant surface decontamination procedure releasing relatively more titanium and metal debris. The surface topography is completely modified after implantoplasty, and the external geometry of the implant is removed (using different drills) to facilitate decontamination. A second procedure (polishing) is performed to reduce the surface roughness. As a consequence, particles of different sizes (nanometric and micrometric) and weights are released. It is recommended to use sufficient irrigation during and after the implantoplasty procedure, as well as powerful surgical suction that will be useful for removing released particles from the peri-implant tissues.

#### 2.3.4. Air-Abrasion

Air-abrasion or polishing is a mechanical method for cleaning teeth and implants surfaces. The method uses powders with different particle sizes contained in a waterjet [152,153] that can remove contaminants and clean and polish the surface [154]. Low-abrasive powders (glycine and erythritol) are used in the waterjet, and a specially designed subgingival nozzle allows application to contaminated implant areas [155,156,157].

The cleaning efficiency of the air-abrasion method was investigated by Tastepe et al. [158]. The authors applied air-abrasion to the simulated subgingival area of titanium implant surfaces in 48 titanium discs. The most efficient decontamination was obtained when the parameters of cleaning were adjusted as follows: application of higher air pressure (while avoiding air emphysema), better insertion of the nozzle tip into the subgingival area, increased movement of the nozzle tip in the subgingival area (up-down, rotation, and slow upward movements) and sufficient water flow [158].

Ronay et al. [159] compared three implant surface debridement methods: curettes, ultrasonic scaling and air-powder abrasion. The authors found that air-abrasion cleaned more surface area than the other methods and did not alter the titanium surface [159]. Duarte et al. [160] evaluated bacterial adhesion on smooth and rough surfaces after decontamination with one of the following procedures: erbium-doped:yttrium, aluminium, and garnet (Er:YAG) laser, metal and plastic curettes and air-powder abrasion. They found that smooth implant surface roughness was increased when metal curettes were used, and rough surfaces showed reduced roughness and bacterial adhesion when metal curettes followed by air-abrasion were used; the authors did not evaluate the presence of metallic debris [160].

Kreisler et al. [161] compared Er:YAG laser and an air-powder system to remove *Porphyromonas gingivalis* from titanium plates. After the surface treatment, fibroblasts were incubated on the specimens, and the proliferation rate was evaluated. They found that both treatments (laser and air powder) supported comparable cell growth, but the air-powder treatment produced slight changes on the implant surface, whereas the laser-treated surfaces remained unchanged [162]. Apparently, in air-abrasion, the optimal air pressure for decontaminating a surface without causing significant alterations is 60–90 psi for 60 s [163].

##### Remarks

Air-abrasion methods with soft powders seem to provide adequate decontamination of titanium discs and titanium implant surfaces. The surfaces treated with this method show minimal or no titanium particle release when the air pressure is adequate (60–90 psi) and sufficient water flow is provided. To reach subgingival areas, specially designed tips are required. This method is apparently safe for dental implant surfaces.

#### 2.3.5. Chemical Decontamination Methods

Chemical methods can be used alone or in combination with mechanical methods for more efficient implant surface decontamination. Chemical methods reduce bacterial adhesion and eliminate bacterial toxins or sub-products present at the implant surface. Among these chemicals are citric acid, tetracycline, saline, chlorhexidine, hydrogen peroxide, tetracycline and doxycycline [164,165,166,167]. Usually, a carrier (cotton swab) is immersed or soaked in the chemical solution and applied to the implant surface with a rubbing movement to clean all the contaminated implant surfaces [168].

Wheelis et al. [168] observed that the combination of rubbing, treatment with a carbon dioxide laser, and any of the following chemicals produced different levels of surface corrosion: citric acid, 15% hydrogen peroxide, chlorhexidine gluconate, tetracycline, doxycycline, sodium fluoride and peroxyacetic acid. Surface corrosion and pitting were presented when more acidic solutions were used (pH < 3); mildly acidic solutions caused surface discoloration, and neutral solutions did not cause signs of corrosion. However, EDS-analysis of all cotton swabs showed the presence of titanium particles [168].

This findings can be explained as follows: acidic solutions (pH < 3) and/or solution with high fluoride concentrations (greater than 0.2%) disrupt the oxide layer on the titanium surface and may inhibit re-passivation (resulting in corrosion), causing localized dissolution of the bulk titanium (pitting), discoloration and the release of ions and metallic debris into the surrounding medium [92,96,129,169,170,171,172,173].

Chemicals for decontamination have also been used in combinations; Wiedmer et al. [174] used combinations of hydrogen peroxide and titanium oxide (H_2_O_2_ + TiO_2_) compared to H_2_O_2_ alone and chlorhexidine (CHX) for the decontamination of titanium surfaces contaminated with *Staphylococcus epidermidis* biofilms. The authors found that surface treatment with a H_2_O_2_ + TiO_2_ suspension was superior to that with H_2_O_2_ and CHX for the decontamination of dental implants. This antimicrobial effect is produced by the chemical interaction of TiO_2_ particles with H_2_O_2_, producing ROS (hydroperoxyl and hydroxyl radicals) and rupturing the bacterial membrane. Unfortunately, its effects on the titanium oxide layer have not been confirmed [174].

Among chemical methods, the application of citric acid is considered slightly superior to that of saline for biofilm removal from titanium surfaces [175,176]. However, studies showed that citric acid at a 40% concentration applied by rubbing to the titanium surface induced changes in the topography and potentially increased the surface roughness; because of its acidic nature, it also potentially dissolves the titanium oxide layer [96,177,178,179].

Ungvari et al. [180], compared three chemical cleaning methods (3% H_2_O_2_ for 5 min; saturated citric acid at pH 1 for 1 min, and chlorhexidine gel at 0.12% for 5 min). After treatment, the samples were evaluated by atomic force microscopy (AFM) and X-ray photoelectron spectroscopy (XPS). AFM showed a slight increase in the surface roughness but did not reveal differences in roughness among the groups, and XPS confirmed the presence of an intact TiO_2_ layer on all the surfaces, thus demonstrating that these chemicals applied under these conditions will not damage the titanium oxide layer of the titanium surface [180].

##### Remarks

Chemical methods facilitate the removal of plaque, elimination of bacteria and reduction of toxins deposited on the implant surface. As a consequence of their pH, some chemical methods can damage the titanium oxide layer and produce corrosion of the titanium surface. When applying chemical substances to an implant surface, the friction removes existing corrosion products, releasing titanium particles from the implant surface and leaving the bulk titanium exposed.

Among the chemical decontamination methods, it seems that saline, chlorhexidine, hydrogen peroxide and saturated citric acid (less than 1 min) produce minimal alterations on the implant surface. There is a lack of information about the effects of tetracycline and doxycycline on titanium particle release from implant surfaces.

#### 2.3.6. Laser Decontamination Methods

The antimicrobial activity of laser light is based on its photothermal effects [181] and its capability to denature proteins [182], and these effects on titanium surfaces are related to the wavelength and operation settings.

When the CO_2_-laser beam is directed onto a titanium implant surface, the laser light is reflected by rougher surfaces [183].

Different power settings for CO_2_ lasers might have different impacts on the titanium surface. The effect of laser irradiation on four titanium surfaces at a power of 1.0 W and 4.0 W with 50 pulses per second (pps) and energies from 15.2 to 60.8 Joules per pulse with a laser beam diameter of 200 μm was compared and surface alterations were found both macroscopically (dark spots) and microscopically (melted and glazed Ti) at power outputs above 2 W [183,184,185,186]. Shibli et al. [187], who evaluated the effects of laser decontamination of the surface of failed titanium implants. A CO_2_-laser at a power of 1.2 W and energy of 40 J for 40 s at a distance of 30 mm from the implant surface was used. No alterations in the implant surface were detected by SEM or EDS analysis [187].

Also Park et al. [188,189] showed that the CO_2_-laser at a low power (1.0 or 2.0 W) did not alter the implant surface, regardless of implant type, while at of 3.5 and 5 W, the laser produced surface alterations and gas. Romanos et al. [190] evaluated the effects of CO_2_ lasers for the treatment of peri-implantitis and concluded that the application of a CO_2_ laser does not alter the implant surface, decreases bacterial contamination, and enhances osseointegration. Finally, Stuebinger et al. [191] compared different lasers for titanium surface decontamination. The CO_2_ laser at a power of 2, 4 and 6 W for 10 s in continuous-wave mode did not modify surfaces [191,192,193].

Schmage et al. [194] compared 10 methods for the surface decontamination of titanium discs. An Er:YAG laser was used in pulsed mode at a distance of 2 mm from the implant surface and the authors found that it produced slight surface damage and only partial bacteria removal. Unfortunately, laser energy, power pulse rate were not reported [194].

The Er:YAG-laser can induce different effects on different implant surfaces. Shin et al. [195] evaluated the effect of Er:YAG-laser irradiation on the microscopic structure and surface roughness of different implant surfaces. Titanium implants with anodized and SLA surfaces were irradiated with an Er:YAG-laser with a 60, 100, 120 and 140 mJ/pulse at 10 Hz. No significant surface changes were observed in SLA surface implants, but severe changes were observed in anodic-oxidized implants with only 100 mJ/pulse irradiation [195].

Er:YAG-lasers seem to be safe for titanium surface at a power of 1 W. Park et al. [189] used higher powers of 1, 2, 3, 4 and 5 W at 20 pulses per second on pure titanium discs (machined and anodized). They observed that 2, 3, 4 and 5 W of power generated melting, coagulation and microfractures of the titanium surface in proportion to the used power.

The time of laser exposure and the energy applied are also responsible for the alterations observed in different titanium implant surfaces. Settings of 100 mJ/pulse, 10 Hz and 1 min preserve the titanium surface structure [196,197]. Meanwhile, higher energies and longer times produce surface melting, particle loosening, and surface fractures on hydroxyapatite (HA)-coated surfaces and TiO_2_ surfaces [198,199]. Finally, Takagi et al. [200] analysed the efficacy of implant decontamination (calcified deposit removal) and the surface alterations of treatment with erbium lasers (Er:YAG and Er,Cr:YSGG), cotton pellets and titanium curettes. The authors found that Er:YAG and Er,Cr:YSGG lasers at 40 mJ/pulse (ED 14.2 J/cm^2^/pulse) and 20 Hz with water spray in non-contact mode were superior to cotton pellets or titanium curettes and the surfaces suffered minimal damage [200].

Romanos et al. [201] analyzed the effect of a diode laser (980 nm) with different power settings applied to titanium discs in continuous-wave mode in comparison with an Nd:YAG laser. Castro et al [202] also showed intact titanium surfaces after treatment with the diode laser but extensive melting and damage after irradiation with the Nd:YAG laser [201,202].

Gonçalves et al. [203] used a 980-nm diode laser with continuous emission for 5 min at 2.5 and at 3 W to irradiate implants contaminated with *P. gingivalis* and *E. faecalis* and evaluated the bacteria reduction and the changes to the implant surfaces. The authors demonstrated that while the used parameters did not change the treated surfaces, 3 W was 100% effective for surface decontamination [203]. The thermal effects of diode lasers (810 nm and 980 nm) when used at low energy (1 W) in pulsed mode with air/water cooling maintained the temperature below the critical threshold of 47 °C [204,205,206].

##### Remarks on Laser-Assisted Decontamination Methods

Lasers used for titanium surface decontamination produce titanium surface alterations depending on the laser energy, radiation time and titanium surface characteristics. There are no studies measuring titanium particles released during or after laser decontamination. The changes in the titanium surface induced by lasers are mainly produced by the temperature increase at the irradiated spot. Each laser behaves differently on rough or machined surfaces. In general, surfaces that reflect the laser (machined surfaces) produce smaller local temperature increases but can affect the surrounding areas. On the other hand, surfaces that absorb the laser (rough surfaces) show significant implant temperature increments. Safe operation settings for each laser and surface should be carefully evaluated before the laser decontamination of implant surfaces to avoid thermal damage and surface changes. To reduce the surface damage during laser decontamination, it is recommended to use pulsed mode, short periods of irradiation, cooling with proper air-water ratios and low energies. This section may be divided by subheadings. It should provide a concise and precise description of the experimental results, their interpretation as well as the experimental conclusions that can be drawn.

## 3. Materials and Methods

PICO questionnaires were developed and problems, interventions, comparisons and outcomes were organized for the surgical, prosthetic and maintenance phases in implant dentistry procedures (Table 2). An exhaustive search of the literature was performed in PubMed, Medline and Google Scholar for all the relevant studies in the literature published between 1980 and 2018 involving titanium particles and ions related to dental implants and implant dentistry procedures. The following search keywords were used in this systematic review.

### 3.1. For the Surgical Phase

Implant bed preparation AND titanium particles; OR implant bed preparation AND metal release; OR bone drilling AND titanium particles; OR bone drilling and metal release; metal debris AND drills; OR implant drill AND wear; OR implant drill and corrosion; OR implant drill and damage; OR bone piezosurgery AND titanium particles/ions; OR osteotomy and metal particles/ions; OR osteotomy and metal debris.

Implant insertion AND metal ions; implant insertion AND titanium ions; Implant insertion AND titanium particles; OR implant insertion and implant surface alterations; OR implant insertion AND titanium release; implant insertion and metal release; OR implant insertion AND titanium particles detachment; OR implant insertion and metal debris; OR implant insertion AND bone contamination; OR implant insertion and bone metal content; implant insertion and tissue metal content.

Implant removal AND metal ions release; implant removal and titanium release; Implant removal AND titanium particles; implant removal and surface alterations; OR implant removal AND metal release; OR Implant removal and particles dislodgement; OR implant removal and metal debris.

### 3.2. For the Prosthetic Phase

Implant abutment connection AND wear; OR implant abutment connection and deformation; OR implant abutment connection AND titanium particles; implant abutment connection AND ions release; OR implant abutment connection AND corrosion; OR implant abutment connection material AND wear; OR implant abutment connection misfit; implant abutment connection misfit AND wear; dental Implants AND fretting corrosion.

### 3.3. For the Maintenance Phase

Implant decontamination AND wear; OR implant decontamination and corrosion; OR fluoride AND titanium corrosion; OR chlorhexidine AND titanium corrosion; OR implant scaling AND titanium wear; OR implant surface polishing AND particles release; implant surface polishing AND titanium findings in soft tissues; implant surface polishing AND titanium particles in bone; OR chemical decontamination AND implant surface OR laser decontamination AND implant surface OR laser decontamination AND titanium particles OR laser decontamination AND titanium ions.

Two investigators (RD and GR) performed the initial searches using the keyword combinations; the titles that appeared in the search containing the keywords were reviewed and these fulfilling the inclusion criteria were included for abstract review. The abstracts of the articles were read in full, and those fulfilling the inclusion criteria were included for full-text review and data extraction.

In the case of a disagreement between reviewers, a third investigator (JLC) was included for a final decision regarding the inclusion or exclusion of the articles.

The inclusion criteria were determined as follows: experimental, animal and human studies published in the English language that analyzed titanium particle or ion release in the surgical, prosthetic/restorative or maintenance phase in implant dentistry. The titanium/metal particles released during implant insertion, particle size, location and detection methods were compiled (Table 3).

The exclusion criteria were as follows: articles in languages other than English, reviews, other systematic reviews and expert opinions as well as duplicated articles were excluded.

## 4. Conclusions

Dental implants have revolutionized the dentistry profession, and titanium dental implants have demonstrated their utility and safety for more than 40 years. However, over time, it has become evident that debris and sub-products can be generated during the life of the implant.

Titanium particles and ions can also be released from metallic instruments used for implant bed preparation, from the implant surfaces during insertion and from the implant-abutment interface during insertion and functional loading. In addition, the implant surfaces and restorations are exposed to the environment, saliva, bacteria and chemicals that can potentially dissolve the titanium oxide layer. If these agents attack continuously, the implant surface can permanently lose its titanium oxide layer.

The formation of soluble compounds on the titanium surface will alter the implant surface chemistry and facilitate the dissolution and degradation of exposed bulk titanium, resulting in the initiation of corrosion cycles. Implant maintenance procedures can potentially alter implant surfaces and produce titanium debris that will be released into the peri-implant tissues.

Multiple variables, such as the bone density, mechanical overloading and the use of fluorides, can also influence the proportion of metal particles and ions released from implants and restorations. The complex oral environment can also change with age and the use of medications, and these factors have not yet been studied.

This review provides, for the first time, a summary of the potential sources of titanium particles and ion release in implant dentistry and, based on the findings, suggests methods for reducing this release. The long-term local and systemic effects of titanium particles and ions released into the oral environment and their potential effects on cells, tissues and organs remain unknown due to the rapid evolution and variability of new implant surfaces, new implant-abutment connections and new restorative materials.

## Figures and Tables

**Table 1:** Titanium/metal particles released during implant insertion. The table summarizes the particle size, locations and detection methods. The particles ranged from a few nanometers to micrometers in size.

Author and Year of Publication	Original Implant Surface	Animal Model and Area of Implant Insertion	Localization of the Metal Particles	Method of Detection	Metal Detected	Particle Size/Recovered Particle Weight	Particle Geometry
Schliephake et al. [43]	Titanium, machined	Minipig mandible	Peri-implant bone and implant surface	- Scanning Electron Microscopy (SEM) - Energy-Dispersive X-ray Spectroscopy (EDX)	Titanium particles	5–30 µm	Solid and leaf-like particles
			Lungs, liver and kidneys	- Flameless atomic absorption spectroscopy (FAAS)	Titanium concentration as ng/mg dry weight of the organ	Kidneys: 2.92 ± 0.69 ng/mg Liver: 11.5 ± 1.35 ng/mg Lungs: 135.7 ± 12.42 ng/mg	-
Tanaka et al. [5]	TPS	Dog mandible	Implant-bone interface and surrounding bone tissue	- SEM - Transmission Electron Microscopy (TEM) - X-ray microanalyzer - Electron diffraction	Titanium particles	1.8–3.2 µm	-
Martini et al. [46]	- TPS - TPS + coating of fluorohydroxyapatite	Mongrel sheep femoral and tibial diaphysis	- Surface of TPS implants - Inside the new bone - Inside the medullary spaces near the TPS surface	(EDS)	Titanium particles	-	-
Franchi et al. [47]	- Titanium, machined - TPS - Alumina oxide, sandblasted and acid-etched - Zirconium oxide, sandblasted + acid-etched	Sheep femur and tibia	- Peri-implant tissue - Inside the new bone - Near blood vessels of peri-implant connective tissue around TPS implants	SEM	Titanium granules	3–60 µm	-
Wennerberg et al. [50]	- Titanium, turned - Titanium, sandblasted	New Zealand rabbit tibia	- Titanium detection was related to the distance of evaluation	- X-ray fluorescence spectroscopy (SRXRF) - Secondary ion mass spectrometry (SIMS)	Titanium concentration as ng/mg dry weight of implant	Turned: 206.7 ± 25.2 ng/mg Sandblasted: 210 ± 35.2 ng/mg	-
Meyer et al. [60]	- Titanium, sandblasted + acid-etched - TPS - Machined	Minipig mandible	- Titanium particles detected at the crestal bone	- SEM - EDS	- Titanium particles and nanoparticles	20 nm to a few microns	- Angular or round elongated particles - Large and oval-shaped particles
Flatebo et al. [55]	- Titanium, anodized	- Humans	- Titanium particles detected in the gingival mucosa around cover screws	- Laser ablation inductively coupled plasma mass spectrometry (LA-ICP-MS) - High-resolution optical darkfield microscopy (HR-ODM) - SEM	- Titanium particles - Titanium isotopes	140–2300 nm	-
Senna et al. [6]	- Titanium, anodized - Titanium, sandblasted + acid-etched	- In vitro bovine ribs	- Titanium particles detected along the implantation site bone walls and cortical layer - Implant surface damage	- SEM with backscattered electron detection (BSD)	- Titanium particles	10 nm to 20 µm	-
Deppe et al. [54]	- Titanium, sandblasted + acid-etched	- Human cadaver edentulous jaws	- Implant surface damage at the apical thread flanks	- SEM	- Areas of the implant surface with loose material, lack of surface characteristics (delamination)	-	-
Sridhar et al. [56]	- Titanium, sandblasted + acid-etched	- Polyurethane foam blocks with different densities	- No particles were detected	- Digital optical microscopy - SEM - X-ray diffraction (XRD)	-	-	-
Deppe et al. [51]	- Four different implants with different surface treatments were compared - Titanium, sandblasted + acid-etched (Ankylos and Straumann) - Acid-etched (Frialit) - Titanium, anodized (Nobel)	- Porcine mandible	- Evaluation of the implant surface damage/changes	- 3D confocal microscopy	- Changes in the surface topography were detected along all the implant surfaces - Major changes were observed at the apical and cervical areas - -Significant destruction of the surface of anodized implants was recorded	-	-
Pettersson et al. [49]	- Titanium, machined implants - Titanium, anodized implants	- Pig jaw bone	- Peri-implant bone	- SEM for the evaluation of implant surface changes - Coupled plasma atomic emission spectroscopy (ICP-AES) for analysis of the released titanium particles	Titanium particles were detected	- Anodized titanium implant with parallel walls 2.80 ± 0.85 µg - Anodized titanium implant, slightly tapered 2.00 ± 0.56 µg - Machined titanium implant, slightly tapered 0.91 ± 0.36 µg	-

**Table 2:** PICO questions used for preparation of the systematic review to identify sources and aetiological factors for titanium particle and ion release.

P (Probelem)	Subgroup		P (Population)	I (Interventions)	C (Comparisons)	O (Outcomes)
The potential sources of titanium particle and ion release are not known or compiled in the literature	Surgical phase	Implant bed preparation	Experimental, animal and human studies	Bone drilling	Implant drills and other implant bed preparation methods	- Metal content in the adjacent bone or soft tissues after implant bed preparation - Tool wear, damage and corrosion
		Implant placement	Experimental, animal and human studies	Implant Insertion	Implant after Insertion	- Implant surface, alterations after insertion - Metal content in the adjacent bone or soft tissues after implant insertion
		Implant removal	Experimental, animal and human studies	Bone drilling	Other methods for implant removal	- Metal content in the adjacent bone or soft tissues after implant removal - Implant surface alterations and corrosion evaluated after implant removal
	Prosthetic phase	Implant abutment connection	Experimental, animal and human studies	Functional load at the implant abutment connection	Type of connection, misfit gap material	- Implant connection frictional damage - Corrosion and particle/ion release at the implant-abutment connection - Metal content in the adjacent bone or soft tissues
	Maintenance phase	Implant cleaning and decontamination techniques	Experimental, animal and human studies	Implant cleaning, disinfection and polishing	Scaling	- Metal content in the adjacent bone or soft tissues after implant cleaning, decontamination or polishing - Implant surface alterations and corrosion evaluated after implant cleaning disinfection and polishing
					Ultrasonication	
					Rubber cups and brushes	
					Air-polishing	
					Lasers	
					Cleaning and antibacterial substances	

**Table 3:** Titanium/metal particles released during implant insertion. The table summarizes the particle size, locations and detection methods. The particles ranged from a few nanometers to micrometers in size.

Author and Year of Publication	Original Implant Surface	Animal Model and Area of Implant Insertion	Localization of the Metal Particles	Method of Detection	Metal Detected	Particle Size/Recovered Particle Weight	Particle Geometry
Schliephake et al. [43]	Titanium, machined	Minipig mandible	Peri-implant bone and implant surface	- Scanning Electron Microscopy (SEM) - Energy-Dispersive X-ray Spectroscopy (EDX)	Titanium particles	5–30 µm	Solid and leaf-like particles
			Lungs, liver and kidneys	- Flameless atomic absorption spectroscopy (FAAS)	Titanium concentration as ng/mg dry weight of the organ	Kidneys: 2.92 ± 0.69 ng/mg Liver: 11.5 ± 1.35 ng/mg Lungs: 135.7 ± 12.42 ng/mg	-
Tanaka et al. [5]	TPS	Dog mandible	Implant-bone interface and surrounding bone tissue	- SEM - Transmission Electron Microscopy (TEM) - X-ray microanalyzer - Electron diffraction	Titanium particles	1.8–3.2 µm	-
Martini et al. [46]	- TPS - TPS + coating of fluorohydroxyapatite	Mongrel sheep femoral and tibial diaphysis	- Surface of TPS implants - Inside the new bone - Inside the medullary spaces near the TPS surface	(EDS)	Titanium particles	-	-
Franchi et al. [47]	- Titanium, machined - TPS - Alumina oxide, sandblasted and acid-etched - Zirconium oxide, sandblasted + acid-etched	Sheep femur and tibia	- Peri-implant tissue - Inside the new bone - Near blood vessels of peri-implant connective tissue around TPS implants	SEM	Titanium granules	3–60 µm	-
Wennerberg et al. [50]	- Titanium, turned - Titanium, sandblasted	New Zealand rabbit tibia	- Titanium detection was related to the distance of evaluation	- X-ray fluorescence spectroscopy (SRXRF) - Secondary ion mass spectrometry (SIMS)	- Titanium concentration as ng/mg dry weight of implant	Turned: 206.7 ± 25.2 ng/mg Sandblasted: 210 ± 35.2 ng/mg	-
Meyer et al. [60]	- Titanium, sandblasted + acid-etched - TPS - Machined	Minipig mandible	- Titanium particles detected at the crestal bone	- SEM - EDS	- Titanium particles and nanoparticles	20 nm to a few microns	- Angular or round elongated particles - Large and oval-shaped particles
Flatebo et al. [55]	- Titanium, anodized	- Humans	- Titanium particles detected in the gingival mucosa around cover screws	- Laser ablation inductively coupled plasma mass spectrometry (LA-ICP-MS) - High-resolution optical darkfield microscopy (HR-ODM) - SEM	- Titanium particles - Titanium isotopes	140–2300 nm	-
Senna et al. [6]	- Titanium, anodized - Titanium, sandblasted + acid-etched	- In vitro bovine ribs	- Titanium particles detected along the implantation site bone walls and cortical layer - Implant surface damage	- SEM with backscattered electron detection (BSD)	- Titanium particles	10 nm to 20 µm	-
Deppe et al. [54]	- Titanium, sandblasted + acid-etched	- Human cadaver edentulous jaws	- Implant surface damage at the apical thread flanks	- SEM	- Areas of the implant surface with loose material, lack of surface characteristics (delamination)	-	-
Sridhar et al. [56]	- Titanium, sandblasted + acid-etched	- Polyurethane foam blocks with different densities	- No particles were detected	- Digital optical microscopy - SEM - X-ray diffraction (XRD)	-	-	-
Deppe et al. [51]	- Four different implants with different surface treatments were compared - Titanium, sandblasted + acid-etched (Ankylos and Straumann) - Acid-etched (Frialit) - Titanium, anodized (Nobel)	- Porcine mandible	- Evaluation of the implant surface damage/changes	- 3D confocal microscopy	- Changes in the surface topography were detected along all the implant surfaces - Major changes were observed at the apical and cervical areas - Significant destruction of the surface of anodized implants was recorded	-	-
Pettersson et al. [49]	- Titanium, machined implants - Titanium, anodized implants	- Pig jaw bone	- Peri-implant bone	- SEM for the evaluation of implant surface changes - Coupled plasma atomic emission spectroscopy (ICP-AES) for analysis of the released titanium particles	Titanium particles were detected	- Anodized titanium implant with parallel walls 2.80 ± 0.85 µg - Anodized titanium implant, slightly tapered 2.00 ± 0.56 µg - Machined titanium implant, slightly tapered 0.91 ± 0.36 µg	-
